# The effects of creatine pyruvate and creatine citrate on performance during high intensity exercise

**DOI:** 10.1186/1550-2783-5-4

**Published:** 2008-02-13

**Authors:** Ralf Jäger, Jan Metzger, Karin Lautmann, Vladimir Shushakov, Martin Purpura, Kurt-Reiner Geiss, Norbert Maassen

**Affiliations:** 1 Increnovo LLC, 2138 E Lafayette Pl, Milwaukee, WI 53202, USA; 2 Department of Sports Physiology, Hannover Medical School, Carl-Neuberg-Str. 1, D-30625 Hannover, Germany; 3 ISME, Weingartenstr. 2, D-64546 Mörfelden-Walldorf, Germany

## Abstract

**Background:**

A double-blind, placebo-controlled, randomized study was performed to evaluate the effect of oral creatine pyruvate (Cr-Pyr) and creatine citrate (Cr-Cit) supplementation on exercise performance in healthy young athletes.

**Methods:**

Performance during intermittent handgrip exercise of maximal intensity was evaluated before (pretest) and after (posttest) 28 days of Cr-Pyr (5 g/d, n = 16), Cr-Cit (5 g/d, n = 16) or placebo (pla, 5 g/d, n = 17) intake. Subjects performed ten 15-sec exercise intervals, each followed by 45 sec rest periods.

**Results:**

Cr-Pyr (p < 0.001) and Cr-Cit (p < 0.01) significantly increased mean power over all intervals. Cr-Cit increased force during the first and second interval (p < 0.01) compared to placebo. The effect of Cr-Cit on force decreased over time and the improvement was not significant at the sixth and ninth interval, whereas Cr-Pyr significantly increased force during all intervals (p < 0.001). Cr-Pyr (p < 0.001) and Cr-Cit (p < 0.01) resulted in an increase in contraction velocity, whereas only Cr-Pyr intake significantly (p < 0.01) increased relaxation velocity. Oxygen consumption measured during rest periods significantly increased with Cr-Pyr (p < 0.05), whereas Cr-Cit and placebo intake did not result in significant improvements.

**Conclusion:**

It is concluded that four weeks of Cr-Pyr and Cr-Cit intake significantly improves performance during intermittent handgrip exercise of maximal intensity and that Cr-Pyr might benefit endurance, due to enhanced activity of the aerobic metabolism.

## Background

Creatine monohydrate supplementation has been found to enhance high intensity intermittent athletic performance [1]. Long-term creatine supplementation increases the effects of resistance training on muscle volume, strength and power [2,3]. Short-term creatine supplementation results in an increase of muscle force and power output during intermittent exercise, even in the absence of resistance training [4,5]. Facilitated muscle phosphocreatine resynthesis [6] and more rapid and efficient recovery periods [5,7] have been stated as proposed mechanisms for this ergogenic effect. However, a majority of studies suggest that creatine supplementation does not improve endurance exercise capacity [8,9]. The effect of creatine supplementation can be highly variable amongst individuals [10] and low initial muscle creatine content has been found to be a prerequisite for maximum ergogenic effects [6,11]. Total muscle creatine concentration can be increased by approximate 20% using a loading dose of 20 g/d for 6 days followed by a maintenance dose of 2 g/d [12]. A more gradual increase can be achieved by 28-days of low dose (3 g/d) supplementation [12], however, studies examining the effect of slowly loading the muscle with creatine (3 g/d for six weeks [13], 1 g/d or 5 g/d for 10 weeks [14]) on exercise performance showed no significant effect over placebo.

Combining creatine, a weak base which is usually ingested in the form of creatine monohydrate, with an acid intended to boosting endurance exercise capacity such as pyruvic acid, could ultimately benefit athletes involved in sports combining endurance and high intensity exercise. High-dose pyruvate intake in combination with dihydroxyacetone can positively influence endurance exercise capacity [15]. Low-dose, short-term supplementation of an inorganic pyruvate salt failed to increase endurance capability measured by the critical power test (8.1 g/d) [16], failed to increase endurance performance, and had no significant effect on energy metabolism during exercise in well-trained cyclists (7 g Ca-pyruvate per day for 7 days) [17], questioning the efficacy of short-term, low-dose pyruvate administration. The delivery of orally ingested pyruvate to the skeletal muscle is probably small due to partial decarboxylation in the stomach and small intestine, and rapid clearance by the liver to be used as a gluconeogenic precursor [17]. However, long-term pyruvate (6 g pyruvate per day for 28 days) intake has been found to increase plasma pyruvate concentration by 60% (n = 3) [18]. Citric acid might be another suitable candidate since citrate supplementation has been found to increase performance in intense exercise lasting between 2 and 50 min, a duration in which the aerobic metabolism becomes more important [19,20]; however, no conclusive evidence exists that citrate supplementation is able to increase endurance exercise capacity.

A recent bioavailability study on one-time creatine pyruvate (Cr-Pyr) and tri-creatine citrate (Cr-Cit) supplementation showed a significant increase in creatine plasma levels with Cr-Pyr in comparison to creatine monohydrate [21]. Two studies investigating endurance exercise capacity of short-term Cr-Pyr supplementation showed mixed results. 7 days of 7 g per day Cr-Pyr supplementation did not beneficially impact endurance capacity or intermittent sprint performance in well-trained cyclists [22], whereas 5 days of 7.5 g per day Cr-Pyr intake increased paddling speed and resulted in decreased lactate concentrations in Olympic canoeists suggesting an increase in aerobic metabolism [23]. A combination of creatine monohydrate and calcium pyruvate was not more effective than creatine monohydrate supplementation to enhancing maximum strength and power during 5-weeks of in-season college football training [24]. High-dose, short-term Cr-Cit supplementation (4 × 5 g Cr-Cit per day for 5 days) has been found to increase anaerobic working capacity (AWC) in healthy physically active women [25] and is able to delay the onset of neuromuscular fatigue during cycle ergometry [26].

This study investigates the effects of 28-days of Cr-Pyr, Cr-Cit or placebo supplementation on endurance capacity and intermittent handgrip power in healthy young athletes using a daily dose intended to slowly load the muscle with creatine (5 g/d of Cr-Pyr or Cr-Cit, equaling approximate 3 g/d of creatine). Intermittent handgrip exercise of maximal intensity is an exercise that combines endurance as well as high intensity elements. This kind of exercise allows investigating the effects of Cr-Pyr and Cr-Cit supplementation to boosting power and endurance exercise capacity.

## Methods

### Subjects

Forty-nine healthy male subjects participated in this study. All subjects in this investigation participated in a familiarization session. During the familiarization session, subjects were informed as to the experimental procedures, completed a personal/medical history form, creatine supplementation history form and signed informed consent statements in adherence with the human subject's guidelines of the American College of Sports Medicine. The study was approved by the Ethical Review Committee of the University of Paderborn. Subject characteristics are presented in Table 1. No subject in this trial was a vegetarian with all subjects reportedly consuming meat in their daily diet. Exclusion criteria on admission were creatine supplementation within a period of 3 months prior to the experiments or the intake of any other nutritional supplement or medication at the time of the study. The subjects were instructed to avoid changes in their diet and training habits during the study.

**Table 1:** Anthropometrical data and blood values after overnight fast

		**Cr-Pyr**	**P Value (pre/post)**	**Cr-Cit**	**P Value (pre/post)**	**Pla**	**P Value (pre/post)**
Age (years)		26.8 ± 3.6		26.7 ± 4.4		26.3 ± 4.5	
Height (cm)		184.4 ± 4.9		182.7 ± 6.2		180.2 ± 5.4	
Bodyweight (kg)	pre	81.7 ± 10.9	< 0.001	78.1 ± 9.0	< 0.001	77.6 ± 7.3	n.s.
	post	83.2 ± 10.7		79.5 ± 9.2		77.7 ± 7.3	
Body Fat (%)	pre	16.7 ± 4.6	n.s.	15.0 ± 4.3	n.s.	15.0 ± 5.2	n.s.
	post	16.6 ± 4.8		14.7 ± 3.9		14.8 ± 7.3	
Forearm circumference (cm)	pre	29.0 ± 2.2	< 0.05	28.1 ± 1.5	< 0.001	28.3 ± 1.2	n.s.
	post	29.7 ± 2.2		28.6 ± 1.5		28.6 ± 1.2	
Creatine (μmol/l)	pre	94.7 ± 5.5	< 0.001	98.8 ± 8.4	< 0.05	93.1 ± 9.0	n.s.
	post	105.1 ± 6.7		108.3 ± 14.1		93.5 ± 8.5	

Means ± SD, n.s. = not significant.

### Study Protocol

A double-blind, placebo-controlled study was performed over a period of 5 weeks. The subjects performed 3 exercise tests at the same time of the day (day 1: incremental test, day 8: pre-test, day 35: post-test).

#### Choice of muscle group and type of exercise

Handgrip exercise is a simple movement with low dependence on coordination. The exercise targets a specific muscle group while the use of auxiliary muscles is excluded. During exercise of this muscle group, blood samples from the working muscles can easily be taken from a cubital vein [27]. The tone of the sympathetic nerve system [28] and the composition of the arterial blood remain almost unchanged. Any change of a parameter of cubital venous blood can almost exclusively be attributed to the working muscles. Thus, differences between the trials can be interpreted as consequences of the different supplementations on muscle metabolism.

#### Incremental test

**Figure 1:**
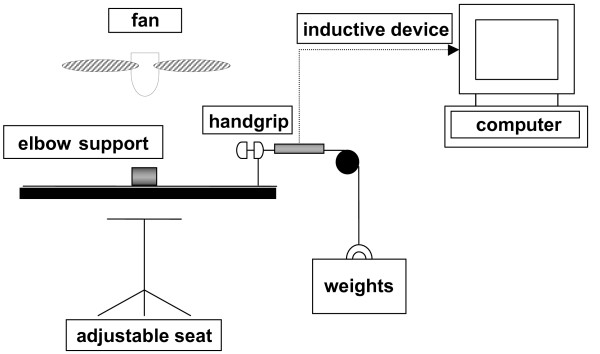
**Schematic presentation of the experimental set up. **The working arm was in a horizontal position supported under the elbow. The handgrip had to be squeezed with the highest contraction frequency possible. The maximum displacement was 3 cm. The fan was used to reduce skin blood flow by cooling.

Handgrip exercise was performed with the arm stretched in a horizontal position. The hand was lying on the handgrip and the arm was supported under the elbow. All subjects had to use the right hand in this exercise. The handgrip was connected to a basket that could be loaded with variable weights. Displacement of the weights -at maximum 3 cm- was recorded through an inductive device connected to the basket (see Figure 1). The seat and the arm support were adjusted for each subject to maintain a constant angle at the shoulder between subjects. Maximum forearm performance was measured starting at a weight of 7.5 kg. The weight was increased by 2.5 kg every 3 minutes until subjective fatigue of the muscle group. The contraction frequency was 24 times per minute. A metronome was placed in sight of the subject to facilitate sustaining the correct contraction frequency.

#### Pre and post-test

**Figure 2:**
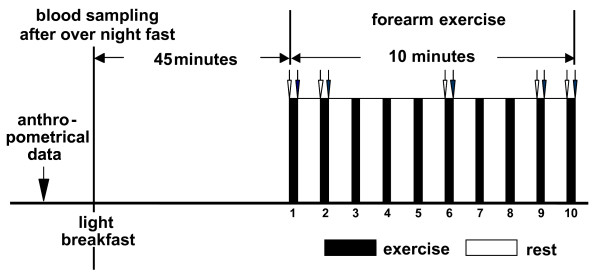
**Forearm exercise started 45 min after the first blood sample had been taken.** Exercise consisted of 10 maximal bouts of 15 sec separated by a 45 sec rest period. Timing of blood samples are indicated by small arrows (open arrow: pre exercise sample; filled arrow: post exercise sample).

After an overnight fast the subjects reported to the lab and their anthropometrical data was taken (age, height, body weight, body fat (measured by bioelectrical impedance analysis) and forearm circumference). Afterwards, blood was sampled from a cubital vein to determine kidney function (creatinine, urea), fat metabolism (free fatty acids, Cholesterol (total, HDL, LDL)) and liver function (gamma GT, OT, PT). Exercise started 45 min after blood sampling and after having a standardized light breakfast consisting of two rolls and jam (see Figure 2).

To test the effects of Cr-Pyr and Cr-Cit the subjects performed intermittent, dynamic handgrip exercise of high intensity. The weight of the basket was 80% of the maximum weight reached in the incremental test. The subjects were instructed to squeeze the handgrip as many times as possible during a 15 sec exercise period. Ten intervals were carried out separated by breaks of 45 sec. A fan was placed above the forearm to reduce skin blood flow by cooling. Blood samples were taken before and after the 1^st^, 2^nd^, 6^th^, 9^th ^and 10^th ^interval from a cubital vein. Blood hemoglobin ([Hb]) and oxy-hemoglobin concentrations ([HbO_2_], OSM III, Radiometer, Copenhagen) as well as hematocrit (Hct, microcentrifugation at 19500 g, Biofuge, Hereaus) were measured. Arterio-venous difference in oxygen (a-vDO_2_) was calculated assuming an oxygen saturation of 95% in the arterial blood. Lactate concentration ([Lac]) was measured polarimetrically in cubital venous blood (Biosen 5030, EKF Barleben, Germany). Ammonia concentration [NH_3_] was determined in plasma (test no. 1877984, Roche, Mannheim, Germany). In order to calculate NH_3_-release it was assumed that arterial [NH_3_] remained constant during the present exercise protocol. The fraction of NH_3 _entering the red cells was neglected. Blood flow was measured by venous occlusion plethysmography during the recovery periods between intervals 6 and 7.

#### Calculation of mechanical data

The first 3 contractions of each interval were discarded because some of the subjects adjusted the position of their hand. Work per contraction (J) was calculated from the displacement and the lifted weight. Total work was calculated from the sum of work of each contraction performed in the first, second, sixth and ninth interval. Mean power was calculated as total work divided by the time evaluated. Contraction velocity was calculated from displacement and contraction time. In the same way, relaxation velocity was calculated. Force was calculated from the contraction velocity and the lifted weight. The 10^th ^interval was excluded from the evaluation as subjects often used additional muscles (movements of upper body) during the last interval.

### Experimental conditions

The subjects were assigned in random order to either the Cr-Pyr (n = 16), the Cr-Cit (n = 16) or the placebo (pla, n = 17) group. It was attempted to match groups for body mass/height ratio and the maximum weight achieved in the incremental test. Athletes with a high degree of handgrip utilization (martial arts, wrestling, etc) could possibly benefit to a greater degree due to training effect, as opposed to sports that neglect the upper extremity/hand grip (track, soccer, etc). To avoid potential differences between groups, athletes with a high degree of handgrip utilization have been equally distributed between groups. The 28-day supplementation period was started immediately after the pre-test and was continued until the day before the post-test. Subjects either received Cr-Pyr (Creapure™ Pyruvate, Degussa, Germany, containing 60% creatine and 40% pyruvate) or Cr-Cit (Creapure™ Citrate, Degussa, Germany, containing 65% creatine and 35% citrate) in form of lemon flavored effervescent tablets at a dose of 5 g per day, while the others received corresponding placebo supplements. Cr-Pyr and Cr-Cit contained <100 ppm creatinine, whilst dicyandiamide and dihyrdotriazine levels and polymeric pyruvates in CrPyr were not detectable by HPLC. The subjects were instructed to take the effervescent tablets (2 in the morning, 1 at noon and 2 in the evening) after the main meals accompanied by a carbohydrate rich drink. Intake of coffee and soft drinks containing caffeine was restricted to two cups a day.

### Statistics

Group data were expressed as mean ± SD and statistical significance was set at the p < 0.05 level. Subjects' characteristics before the investigation were compared using ANOVA. Pre and Post differences of anthropometric data and total means were analyzed by two-way ANOVA for repeated measures with post-hoc t-test considering the multi-comparison problem (Holm-Sidak). Force and relaxation velocity was analyzed by three-way ANOVA. In case of significant main effects for the treatment the effects within the groups were separately analyzed by a two-way ANOVA with repeated measurement in both factors.

## Results

### Anthropometric data and blood parameters

Body weight increased in both creatine groups by a similar amount (p < 0.001, Table 1), whereas the weight in the placebo-group remained unchanged. Circumference of the forearm increased significantly in both creatine groups (p < 0.01, Table 1). Cr-Pyr and Cr-Cit were well tolerated. Creatinine levels increased significantly in both creatine groups but remained within the normal range. Data for all other measured blood parameters were in the normal range and no difference between groups could be determined.

### Performance data

**Figure 3:**
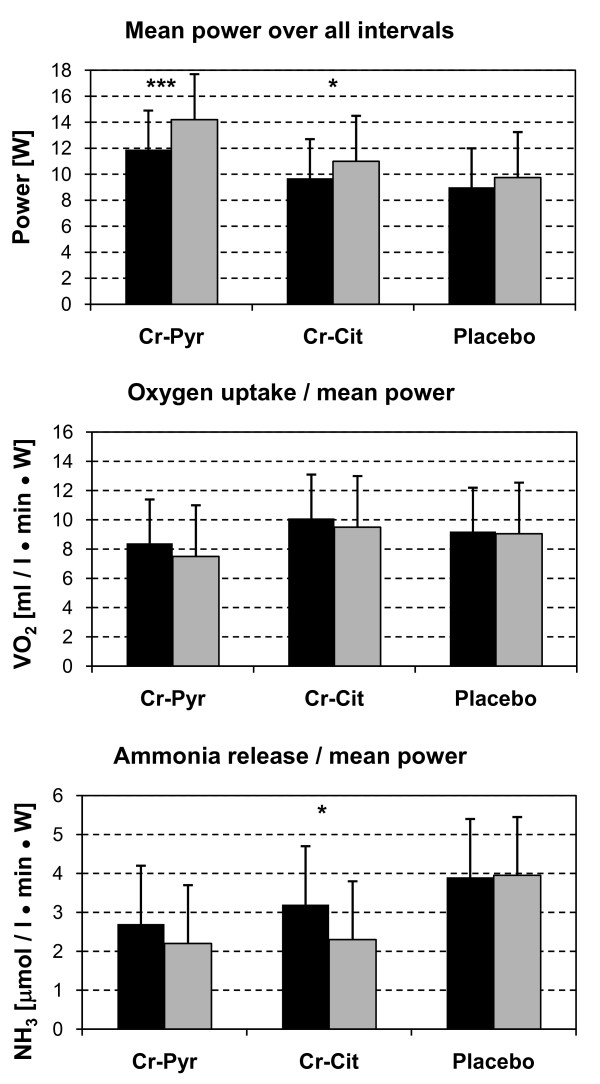
Top: Mean power over all evaluated intervals was significantly improved with Cr-Pyr and Cr-Cit; middle: ratio of mean oxygen to mean power; bottom: Cr-Cit significantly improves ratio mean ammonia release to mean power (Left: pre supplementation, right: post supplementation, *** P < 0.001; * <0.02).

The pre-test performance data was not statistically different among all groups. Supplementation with Cr-Pyr (p < 0.001) and Cr-Cit (p < 0.02) resulted in a significant increase in mean power (see Figure 3), while the placebo group showed no increase in power. A-vDiff in O_2 _increased insignificantly (p > 0.05) in both creatine groups. The oxygen uptake to mean power ratio was slightly reduced with Cr-Cit and Cr-Pyr, however, the changes were not statistically significant (p > 0.05, Figure 3). Blood flow was enhanced with Cr-Pyr but decreased slightly with Cr-Cit, however, both were not statistically significant (p > 0.05). A-vDiff in O_2 _and blood flow measured during the recovery periods significantly increased (p < 0.05) in the Cr-Pyr group concordantly with muscular oxygen consumption compared to the pre-tests. Venous blood lactate levels remained unchanged with either treatment. Mean muscular NH_3 _release was significantly reduced in the Cr-Cit group (p < 0.05). The NH_3 _release per unit of power decreased after Cr-Pyr and Cr-Cit treatments, conversely, only Cr-Cit group resulted in significant changes (p < 0.015, Figure 3).

The contraction velocity increased 24% with Cr-Pyr (p < 0.01) and 20% with Cr-Cit (p < 0.01, Table 2). This increase is accompanied by a significant increase in force (p < 0.001) and relaxation velocity (p < 0.01) in the Cr-Pyr group. The Cr-Cit group showed a significant increase in force (p < 0.01), however, the change in relaxation velocity was not significant. In addition, Cr-Cit significantly increased force only during the first two intervals, when compared to placebo. The effect of Cr-Cit on force decreased over time and the improvement was not significant at interval 6 and 9. The increase in contraction frequency is the major reason for the increase in mean power.

**Table 2:** Force and relaxation velocity.

		**Interval 1**	**Interval 2**	**Interval 6**	**Interval 9**
**Cr-Pyr**					

**Force (N)**	pre	209 ± 47	202 ± 47	202 ± 50	204 ± 52
	post	218 ± 48**	212 ± 49**	212 ± 52**	213 ± 49**
**Relaxation Velocity (m/s)**	pre	0.151 ± 0.033	0.141 ± 0.032	0.143 ± 0.046	0.150 ± 0.040
	post	0.164 ± 0.019*	0.158 ± 0.025*	0.163 ± 0.034*	0.166 ± 0.032*

**Cr-Cit**					

**Force (N)**	pre	191 ± 53	184 ± 51	184 ± 54	184 ± 55
	post	198 ± 54*	193 ± 54*	188 ± 54^‡^	188 ± 55^‡^
**Relaxation Velocity (m/s)**	pre	0.154 ± 0.020	0.135 ± 0.033	0.135 ± 0.033	0.137 ± 0.035
	post	0.169 ± 0.034^‡^	0.142 ± 0.023^‡^	0.135 ± 0.026^‡^	0.140 ± 0.027^‡^

**Placebo**					

**Force (N)**	pre	180 ± 45	173 ± 43	171 ± 44	171 ± 39
	post	182 ± 42^‡^	176 ± 41^‡^	173 ± 38^‡^	174 ± 39^‡^
**Relaxation Velocity (m/s)**	pre	0.155 ± 0.031	0.139 ± 0.036	0.131 ± 0.048	0.146 ± 0.062
	post	0.160 ± 0.032^‡^	0.142 ± 0.037^‡^	0.146 ± 0.047^‡^	0.152 ± 0.049^‡^

Means ± SD, ** p < 0.001, * p < 0.01, ^‡ ^= not significant.

## Discussion

### Main findings

The results of this study suggest that 4 weeks of low dose Cr-Pyr and Cr-Cit intake significantly increased mean power in comparison to placebo. Cr-Pyr improved contraction velocity and reduces fatigability during intermittent exercise of high intensity. Cr-Pyr showed significant improvements in force during all intervals, whereas the effects of Cr-Cit decreased and improvements were not significant during the later intervals. The effect of Cr-Pyr resulted from an increased contraction and relaxation speed and is accompanied by enhanced oxygen consumption and blood flow.

### Effects on body weight

Daily creatine intake ranged from 0.027 to 0.047 g/kg bw (mean 0.037) in the Cr-Pyr group and from 0.036 to 0.052 g/kg bw (mean 0.044) in the Cr-Cit group. Despite a 19% difference in mean creatine dosage per body weight there was a similar increase in body weight in both creatine groups without significant changes in body fat. Weight increased in 15 of 16 subjects (94%) with Cr-Pyr and in 14 of 16 subjects (88%) with Cr-Cit. The weight gain is similar to previously reported weight gains of short-term, high-dose [6,29] or long-term, low-dose creatine supplementation [12].

### Performance enhancement during the first interval

The efficacy of creatine during the first exercise interval is dependent on the type, the intensity and the duration of exercise. The exercise must be of high intensity and of a certain duration allowing PCr stores to drop significantly. Additionally, the efficacy of creatine supplementation seems to be dependent on the speed of movement. During isometric exercise, creatine supplementation was found to have no or small effects on isometric torque production [30,31]. An increase in power of about 20% was measured in fast movements using Wingate tests [32] and isokinetic tests resulted in moderate improvements of mean and peak power (6% and 8%, respectively) [33]. Cr-Cit and Cr-Pyr supplementation increase performance during the first intervals (see Table 2) in contrast to previously published data [5,34]. The increase of Cr-Pyr group was larger compared to the Cr-Cit group; however, the difference was not significant. The type of exercise used in this study consisted of a large amount of negative work. If only the active work is considered (contraction velocity, as the reduction of the displacement can be neglected in the first interval), the improvement is 18 and 15%, respectively, an increase comparable to studies using Wingate tests.

The increase in performance in both creatine groups at the beginning of the intermittent exercise is related to an increased contraction speed. Contraction speed during fatiguing exercise seems to be reduced when free ADP increases [35]. Creatine has been reported to increase ATP re-synthesis and thus reduce the increase in free ADP [36]. This is confirmed by smaller muscular NH_3 _release during exercise which suggests a lower adenosine (AMP) to inosine (IMP) conversion rate (ATP dependent), and thus lower inorganic phosphate concentration. A reduced NH_3 _release or concentration in blood was shown in previous creatine supplementation studies [33,37]. Creatine supplementation results in reduced hypoxanthine concentrations [37] which might be the explanation for the observed improvement in the first interval with Cr-Cit and Cr-Pyr. Relaxation speed increased in both Creatine groups after the first exercise interval comparable to previous observations [31]. But the increase was only significant with Cr-Pyr.

### Performance during repeated intervals

The effect of creatine supplementation on performance in repeated intervals varies with the diversity of tests and the beneficial effect decrease at later intervals of the exercise. Short-term, high-dose creatine supplementation did show the greatest improvement in performance between intervals 4 and 7 during 10 intervals of 6 sec maximum cycling exercise [34]. Subsequent intervals showed a decrease in improvement [34]. Thirty sec exercises intervals resulted in a decrease of improvement in performance from the second interval [36], showed no improvement in the third interval [33], or no difference in the fourth and fifth interval [5]. The beneficial effect of creatine intake on interval exercise was related to an increased rate of PCr re-synthesis during breaks [6,38] but the existing literature is inconclusive. PCr re-synthesis was found not to be enhanced [38] or even reduced [39] and the rate of synthesis defined as dPCr*s^-1 ^after dynamic [38] and isometric work [31] was not influenced by creatine supplementation. Cycling with maximal intensity for 30 sec resulted in decreased PCr levels of 49 mmol kg^-1 ^dry weight without and 57 mmol kg^-1 ^dry weight with creatine ingestion [36]. However, the difference in re-synthesis after exercise was only 2 mmol after 4 min [36] indicating an enhanced accumulation of inorganic phosphate (Pi) during repeated intervals, especially if the breaks are short. Increased Pi might cause fatigue [40] which might be compensated by an increased PCr pool during early intervals. PCr decreases during later intervals [33] and is not able to compensate the effects of increased Pi. The decrease in performance relative to the initial performance might be greater after creatine supplementation [5,31,33,36]. Cr-Cit intake showed such a performance pattern (see Table 2) indicating that fatigue occurred in parallel to PCr-breakdown [41]. If PCr re-synthesis would have been increased after creatine ingestion, Pi concentration should not increase. As a result, ATP production must be enhanced resulting in an increased oxygen uptake during the breaks. Comparable to previous research did Cr-Cit supplementation not change oxygen uptake. Therefore the ratio VO_2 _to mean power decreased slightly (see Figure 3), indicating that the additional energy was derived from anaerobic sources and that PCr re-synthesis was not enhanced.

The increase in performance with Cr-Pyr during the first intervals is slightly larger than with Cr-Cit. Moreover, Cr-Pyr reduces fatigability during all following intervals. The improvement results from an increase in contraction velocity during all intervals and an increase in relaxation velocity [31]. This suggests ADP or Pi levels are reduced and ATP re-synthesis is increased during exercise, which should lead to an increased VO_2_. In fact, the improvement in performance is accompanied by an increase in muscular VO_2 _measured during the breaks. As blood flow decreases during the 15 sec of exercise due to the high forces occurring [42], muscular oxygen uptake during exercise cannot be very high. Therefore, measurements during the breaks are representative for the oxygen consumption during the exercise period. The ratio VO_2 _to mean power drops similar to Cr-Cit indicating that most of the improvement is due to the increased VO_2_, however a small amount of energy is still derived from anaerobic sources. The energy must be derived from phosphate stores since lactate levels did not change. The amount is comparable to that observed after the Cr-Cit supplementation. The additional amount of aerobic work after Cr-Pyr supplementation compensates the creatine effect on ammonia release as the ratio ammonia release to mean power is not significantly different between pre and posttests. Thus the improvement by Cr-Pyr seems to be caused by a different mechanism perhaps resulting in increased ADP concentration compared to Cr-Cit.

### Possible reasons for the increased aerobic metabolism

A potential creatine effect on the time course of VO_2 _is related to Phosphocreatine breakdown (Bessman cycle or Phospho-Creatine shuttle) [43]. Cr-Cit and Cr-Pyr supplementation should result in a similar increase in muscle creatine content; hence varieties in VO_2 _cannot be explained by differences in the Bessman cycle. Additionally, there are some studies showing that VO_2 _during exercise is unaffected [9], or the VO_2_-kinetics is even slowed down after creatine supplementation [44] and oxygen consumption after exercise of high intensity or during breaks is unchanged [45].

The observed results suggest additional pyruvate benefits in the Cr-Pyr group. The role of pyruvate supplementation might be to decrease the relative inhibition on aerobic glycolysis due to elevated creatine phosphate and ATP levels. It may also be reflective of a more rapid regeneration of ATP and reduction in the inorganic phosphate increase, allowing for higher intracellular calcium ion concentration, permitting the higher frequency contraction and relaxation. To achieve an additional effect of pyruvate an increase in plasma pyruvate concentration should occur. A recent study showed that one-time administration of 5 g Cr-Pyr does not increase plasma pyruvate concentrations [21]. However, previous research found a cumulative effect of pyruvate administration increasing the plasma pyruvate concentration by about 60% after 4 weeks [18]. In the intestine and the liver, pyruvate can easily be converted to ketone compounds [46] or different amino acids [47]. If parts of these substances or of the pyruvate absorbed were distributed to the muscle the pool of substances for anaplerotic reactions would increase. This enables the muscle to replenish the pool of tri-carbon-cycle intermediates rapidly at the beginning of exercise. This replenishment might favor the flux through the TCA cycle and thus increase the delivery of protons to the respiratory chain. The increased concentration of intermediates might also act as a pyruvate buffer, with the function to deliver carbon skeletons to the TCAC. Both mechanisms could be beneficial during the transition from rest to high intensity exercise to increase oxygen uptake [48]. Given the transient presence of anaplerotic substrates, the effect might be short-lived and not subject to long-term accumulation over time. The results of more acute administration or different organic salts of pyruvate would be of interest.

## Conclusion

Cr-Cit and Cr-Pyr supplementation significantly increases mean power in high intensity exercise. Cr-Pyr intake significantly increases force and decreases fatigability during all intervals due to an enhanced contraction and relaxation velocity. The performance with Cr-Cit decreases with time and improvements were not significant during the later intervals. Further research is required to exam the mechanism of decreasing fatigue during intermittent exercise of high intensity observed with Cr-Pyr intake.

## Competing interests

The author(s) declare that they have no competing interests.

## Authors' contributions

NM, KRG, RJ and MP participated in the design of the study. NM, JM, KL and VS organized the blood collection and assayed the samples. NM analyzed the results statistically, and RJ and NM drafted the manuscript. All authors have read and approved the final manuscript.
